# Draft Genome Sequence of the Type Strain *Pseudomonas umsongensis* DSM 16611

**DOI:** 10.1128/genomeA.01038-17

**Published:** 2017-09-28

**Authors:** Ewa M. Furmanczyk, Michal A. Kaminski, Andrzej Dziembowski, Leszek Lipinski, Adam Sobczak

**Affiliations:** a Institute of Biochemistry and Biophysics, Polish Academy of Sciences, Warsaw, Poland; b Institute of Genetics and Biotechnology, Faculty of Biology, University of Warsaw, Warsaw, Poland

## Abstract

Here, we report the draft genome sequence of *Pseudomonas umsongensis* type strain DSM 16611. The assembly consists of 14 contigs containing 6,701,403 bp with a GC content of 59.73%.

## GENOME ANNOUNCEMENT

The genus *Pseudomonas* contains microorganisms that can degrade a wide range of xenobiotics, such as phenol, trinitrotoluene, xylene, polyaromatic hydrocarbons, and petroleum. Here, we present the draft genome sequence of *Pseudomonas umsongensis* type strain DSM 16611 (= Ps 3-10 = LMG 21317^T^ = KACC 10847^T^ = CIP 108618^T^), isolated from agricultural soil in South Korea (1). A member of this species—strain Gwa3, isolated from oil-contaminated soil—is a psychrophilic bacterium that has the ability to degrade petroleum hydrocarbons efficiently (2). The primary degradation of these compounds is mediated by enzymes called oxygenases and is also enhanced by the production of biosurfactants that increase the solubilization of hydrocarbons (2).

Genomic DNA was isolated according to a previously described protocol (3). Illumina paired-end (with an average insert size of 300 bp) and Nextera mate pair (with an average insert size of 8 kb) libraries were prepared according to the manufacturer’s instructions (with a KAPA HTP DNA library preparation kit for Illumina sequencing and a Nextera mate pair sample prep kit, respectively). Whole-genome sequencing of *P. umsongensis* DSM 16611 was performed using the Illumina MiSeq platform (2 × 300 bp) and resulted in 912,509 paired reads for the paired-end library and 2,006,908 paired reads for the mate pair library. Reads from the paired-end library were processed as follows: adapters were removed using the Cutadapt script (4), the reads were filtered by length (>100) and quality (q30) (5), and only paired reads were used for assembly. The mate pair reads were processed with NxTrim (6), and only real mate pair reads were used for the assembly. Assembly was done using SPAdes version 3.9.0 (7). Contigs longer than 1 kb were deposited in GenBank and annotated using the NCBI Prokaryotic Genome Annotation Pipeline (8). No plasmid sequence was detected in the draft genome sequence.

The assembly consisted of 14 contigs containing 6,701,403 bp, with a GC content of 59.73%. The DSM 16611 genome encodes 6,152 predicted genes, from which 5,937 are protein coding, and has 73 RNA genes (62 tRNAs, 7 rRNAs, 4 noncoding RNAs) and 142 pseudogenes.

The genome contained genes potentially involved in both protection from chemical compounds and active decomposition of them. The analyzed genome contained open reading frames for proteins engaged in the synthesis of rhamnolipid (*rfbADB* and *rfbC*), one of the biosurfactants with a glycolipid structure that is produced by several *Pseudomonas* spp. This biosurfactant could be significant in the emulsification of the hydrocarbons during degradation. The DSM 16611 genome encompassed genes encoding enzymes known to be involved in the degradation of oil components, including amino benzoate (*ant* genes) and benzoate (*pca* and *ben* genes) metabolism. Moreover, many oxygenase genes have been identified in the DSM 16611 genome (including methane monooxygenases and heme monooxygenases), possibly involved in the decomposition of alkanes, alkenes, and cycloalkanes.

### Accession number(s).

This whole-genome shotgun project has been deposited at DDBJ/ENA/GenBank under accession no. NIWU00000000. The version described in this paper is the first version, NIWU01000000.
